# Diverse range dynamics and dispersal routes of plants on the Tibetan Plateau during the late Quaternary

**DOI:** 10.1371/journal.pone.0177101

**Published:** 2017-05-05

**Authors:** Haibin Yu, Yili Zhang, Zhaofeng Wang, Linshan Liu, Zhao Chen, Wei Qi

**Affiliations:** 1 Key Laboratory of Land Surface Pattern and Simulation, Institute of Geographic Sciences and Natural Resource Research, Chinese Academy of Sciences, Beijing, P. R. China; 2 School of Life Sciences, Sun Yat-sen University, Guangzhou, P. R. China; 3 University of Chinese Academy of Sciences, Beijing, P. R. China; 4 Guangdong Institute of Eco-environmental Science & Technology, Guangzhou, P. R. China; National Cheng Kung University, TAIWAN

## Abstract

Phylogeographical studies have suggested that several plant species on the Tibetan Plateau (TP) underwent recolonization during the Quaternary and may have had distinct range dynamics in response to the last glacial. To further test this hypothesis and locate the possible historical dispersal routes, we selected 20 plant species from different parts of the TP and modeled their geographical distributions over four time periods using species distribution models (SDMs). Furthermore, we applied the least-cost path method together with SDMs and shared haplotypes to estimate their historical dispersal corridors. We identified three general scenarios of species distribution change during the late Quaternary: the ‘contraction-expansion’ scenario for species in the northeastern TP, the ‘expansion-contraction’ scenario for species in the southeast and the ‘stable’ scenario for widespread species. During the Quaternary, we identified that these species were likely to recolonize along the low-elevation valleys, huge mountain ranges and flat plateau platform (e.g. the Yarlung Zangbo Valley and the Himalaya). We inferred that Quaternary cyclic glaciations along with the various topographic and climatic conditions of the TP could have resulted in the diverse patterns of range shift and dispersal of Tibetan plant species. Finally, we believe that this study would provide valuable insights for the conservation of alpine species under future climate change.

## Introduction

Phylogeography has provided a way to understand the demographic history of species, which was primarily affected by periodic climate oscillations during the Quaternary, generating varying degrees of range contraction and expansion [1]. In general, species retreated to refugia during the glacial period, preserving multiple and original haplotypes within these regions [2]. During the postglacial period, several species recolonized in other areas, producing nearly complete haplotype uniformity in the colonized populations [3]. Hence, the range dynamics of species during the Quaternary may have influenced the present-day genetic structures and phylogeographical patterns [4, 5].

Typically, phylogeographers can use mismatch distribution analysis or neutral test to detect whether species underwent recent demographic expansion [6–8], and occasionally to estimate the expansion time based on the mutation rate of DNA sequences [7]. However, the detailed changes in range during the late Quaternary and expansion routes in a spatial context have rarely been identified. Fortunately, the available paleoclimate data and species distribution models (SDMs) have been widely applied to determine the trends in species distribution during the late Quaternary [9–11]. Furthermore, the least cost path method, which is often used in the field of landscape ecology [12–14], has been used to locate past dispersal routes in combination with population genetic data and SDMs [15, 16]. Such integrative calculation would undoubtedly supplement the demographic history of species.

The Tibetan Plateau (TP) has become a hotspot of phylogeographical studies [17–20], owing to its complicated alpine conditions (e.g. climate and topography) and past environmental changes (e.g. Quaternary climate fluctuations). Based on the demographic history of plant taxa inferred from these studies, we found that the changes in distribution were diverse during the late Quaternary. Because there was no formation of large and whole ice sheet during the Last Glacial Maximum (LGM) period on the TP [21], most evidence has shown that the LGM had a limited effect on species distribution [17, 22–24]. Thus, the distributions of numerous species remained stable status throughout the LGM. However, a few species in the northeastern part of the TP, including *Juniperus przewalskii* [25] and *Picea crassifolia* [26], still underwent the typical *tabula rasa* scenario. In contrast, several cold-adapted species (e.g. *Picea likiangensis* and *Taxus wallichiana*) in the southeastern TP experienced range expansion during the LGM [10, 11]. Based on the above-mentioned inferences, we thereby recommend three common patterns of range dynamics across the TP: i) a contraction-expansion scenario for species in the northeastern TP; ii) an expansion-contraction scenario for species in the southeastern TP; and iii) a stable scenario for widespread species throughout the TP. This assumption is only based on the phylogeography of a few species; thus, to further verify this speculation, more species and distribution modelling needs to be carried out.

Several plant species on the TP underwent rapid and extensive recolonization events during the Quaternary [27, 28]. We assumed that these species might be susceptible to climatic fluctuations, we thus chose them in this study to i) test whether species in different parts of the TP had distinct range dynamics during the late Quaternary, and ii) identify the possible historical dispersal corridors of plant species on the TP.

## Data and methods

### Study area

**Fig 1:**
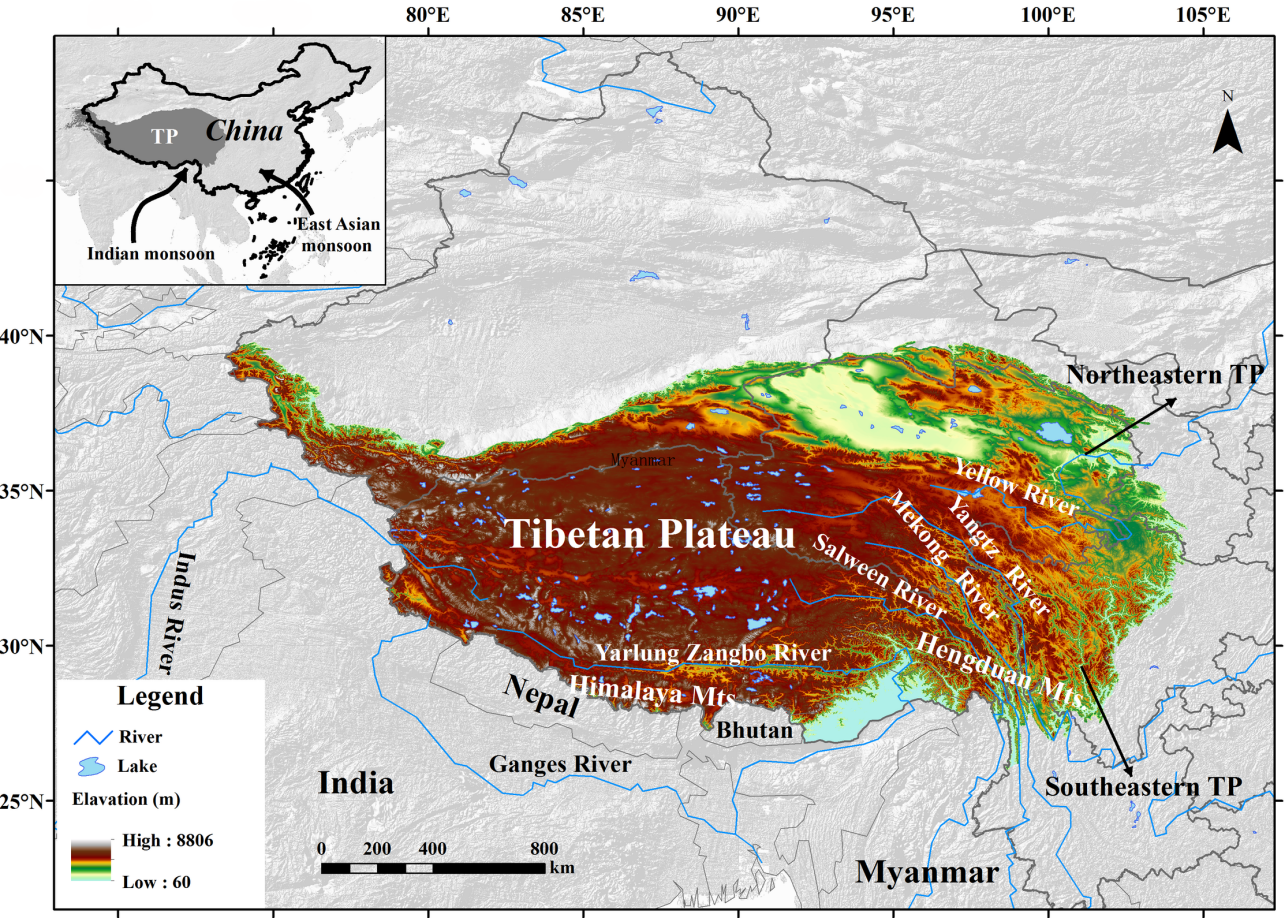
The scope of the Tibetan Plateau and its basic geographical conditions. In this study, the area of northeastern TP includes the Hehuang Valley, the Qilian Mountains and the Qaidam Basin in Qinghai Province, the Taohe Valley in Gansu Province and the Minshan in northern Sichuan Province; the southeastern TP mainly covers the East Himalaya and Hengduan Mountains.

The TP is the largest (*c*. 2.5×10^6^ km^2^) and highest plateau in the world with an average elevation of greater than 4000 m [29] (Fig 1). Several major Chinese and Asian rivers originate from the TP, such as the Yangtze River, Yellow River, Mekong River, Salween River, Yarlung Zangbo River, Indus River and Ganges River. These rivers could be important corridors for the dispersal of water vapor, plants and animals [16, 30]. During the summer season each year, both the Indian and East Asian monsoon bring warm and wet air that is critical for the growth and reproduction of plant species on the TP [31]. In the southeastern TP, the East Himalaya and Hengduan Mountains is a key biodiversity hotspot harboring a great number of endemic and alpine species [32], which is regarded as the potential recent origin areas and Quaternary refugia of many temperate plants of East Asia [33–35].

### Species selection and determination of shared haplotypes

To test whether species in different parts of the TP have distinct range dynamics, we chose 20 plant species from three regions of the TP (Table 1), which experienced demographic expansion during the Quaternary. Based on the phylogeographical study of each species, we collected the haplotype composition and geographical coordinate of each population. To ensure the congruence in DNA markers, we mainly selected maternally inherited markers (e.g. cpDNA for angiosperms and mtDNA for gymnosperms). From the spatial haplotype structures of each plant (S1 Fig), we obtained which populations had shared haplotypes.

**Table 1:** Twenty plant species in different parts of the TP were used in this study.

Species	Family	DNA Type	Population No.	Haplotype No.	Shared Haplotype No.	Reference
**Northeast TP**						
*Juniperus przewalskii*	Cupressaceae	cpDNA	20	6	5	[25]
*Picea crassifolia*	Pinaceae	mtDNA	32	9	9	[26]
*Metagentiana striata*	Gentianaceae	cpDNA	14	10	2	[36]
*Angelica nitida*	Apiaceae	cpDNA	16	20	5	[37]
*Bupleurum smithii*	Apiaceae	cpDNA	22	25	9	[38]
**Southeast TP**						
*Buddleja crispa*	Loganiaceae	cpDNA	23	13	4	[39]
*Picea likiangensis*	Pinaceae	mtDNA	42	9	7	[40]
*Taxus wallichiana*	Taxaceae	cpDNA	43	29	11	[11]
*Spenceria ramalana*	Rosaceae	cpDNA	19	17	6	[41]
*Quercus aquifolioides*	Fagaceae	cpDNA	58	36	11	[42]
**Entire TP**						
*Potentilla fruticosa*	Rosaceae	cpDNA	52	54	17	[22]
*Allium przewalskianum*	Liliaceae	cpDNA	48	32	13	[23]
*Sibiraea angustata*	Rosaceae	cpDNA	38	8	6	[43]
*Hippuris vulgaris*	Hippuridaceae	cpDNA	47	8	4	[44]
*Ranunculus bungei*	Ranunculaceae	cpDNA	44	6	4	[45]
*Pomatosace filicula*	Primulaceae	cpDNA	24	7	5	[46]
*Orinus thoroldii*	Poaceae	cpDNA	28	7	5	[47]
*Rosa sericea*	Rosaceae	cpDNA	61	37	18	[27]
*Stuckenia filiformis*	Potamogetonaceae	cpDNA	54	13	5	[48]
*Anisodus tanguticus*	Solanaceae	cpDNA	32	6	4	[28]

### Modelling species distribution

We applied the available bioclimatic and occurrence data to model the species distribution. Based on the WorldClim database (http://www.worldclim.org/) [49], we obtained 19 bioclimatic variables (S1 Table) with a resolution of 30 s (the data from the LGM were resampled to 30 s) for the Last Interglacial (LIG, *c*. 140–120 ka), the Last Glacial Maximum (LGM, *c*. 22 ka) and the Mid Holocene (*c*. 6 ka) based on the Community Climate System Model (CCSM4), and the current conditions (*c*. 1960–1990). We then clipped these variables according to the approximate species range in ArcGIS 10.1 (Environmental Systems Research Institute, Inc., Redlands, CA, USA). Next, to eliminate highly correlated factors, we carried out an autocorrelation test for 19 clipped variables using the SDMtoolbox v1.1c [50] in ArcGIS 10.1 (ESRI). Consequently, several bioclimatic variables of the four periods with low Spearman’s coefficients (*r* < 0.75) were retained for subsequent analysis.

The species occurrence data (S2 Table) were mainly collected from published literatures and further supplemented with data from field surveys and online database, including the Global Biodiversity Information Facility (GBIF, http://data.gbif.org) and the Chinese Virtual Herbarium (CVH, www.cvh.org.cn). As spatial clusters of localities in the gathered occurrence data may result in model’s inflation and over-fit towards environmental biases [51–53], we applied a spatially rarefy method to filter the occurrence data [53]. Based on the climatic heterogeneity, localities of occurrence were spatially filtered at 5, 15 and 25 km^2^ in areas of high, medium and low climatic heterogeneity, respectively. To calculate climatic heterogeneity, we conducted a principal component (PC) analysis of 19 bioclimatic variables and then calculated the mean standard deviation of the first three climate PCs. These analyses were implemented using the SDMtoolbox v1.1c [50]. Finally, the remaining localities combined with low-correlated bioclimatic layers were used to estimate species distribution.

In this study, we used the maximum entropy algorithm in MAXENT v3.3 [9] to model the current and past distributions of 20 species with a convergence threshold of 10^−5^, 500 iterations, and the localities of occurrence divided into testing data sets and training data sets (25% and 75%, respectively). We then conducted simple statistics on data distribution of range dynamics for each species and different regions in R 3.2.5 [54].

### Visualizing the dispersal routes

We identified the dispersal routes of plant species on the TP under the least-cost path (LCP) method using the SDMtoolbox v1.1c [50] in ArcGIS 10.1 (ESRI). (i) We first obtained a dispersal cost layer (resistance layer) by inverting the species distribution layer (1-SDM) and then created a cost distance raster for each sample locality based on the resistance layer. (ii) Using the cost distance raster, we obtained the corridor layers between two localities with shared haplotype. (iii) In contrast to previous studies [12, 55], which generally obtained linear dispersal routes, we applied the categorical LCP method to better represent environmental heterogeneity in dispersal. We classified the value of each corridor layer into four intervals (three cutoff values); if the lowest value of corridor layer was hypothesized to be 1.00, the four intervals were: 1.00 ~ 1.01 (1.00 + 1.00*1%), 1.01 ~ 1.02 (1.00 + 1.00*2%), 1.02 ~ 1.05 (1.00 + 1.00*5%) and 1.05 ~ highest value. These four intervals were then reclassified as new values (5, 2, 1, 0, respectively). (iv) We summed all of the pairwise reclassified corridor layers, and the spatial dispersal routes for each plant were obtained. Finally, based on the routes of 20 species, we identified the possible recolonization pathways of species on the TP during the Quaternary.

## Results

**Fig 2:**
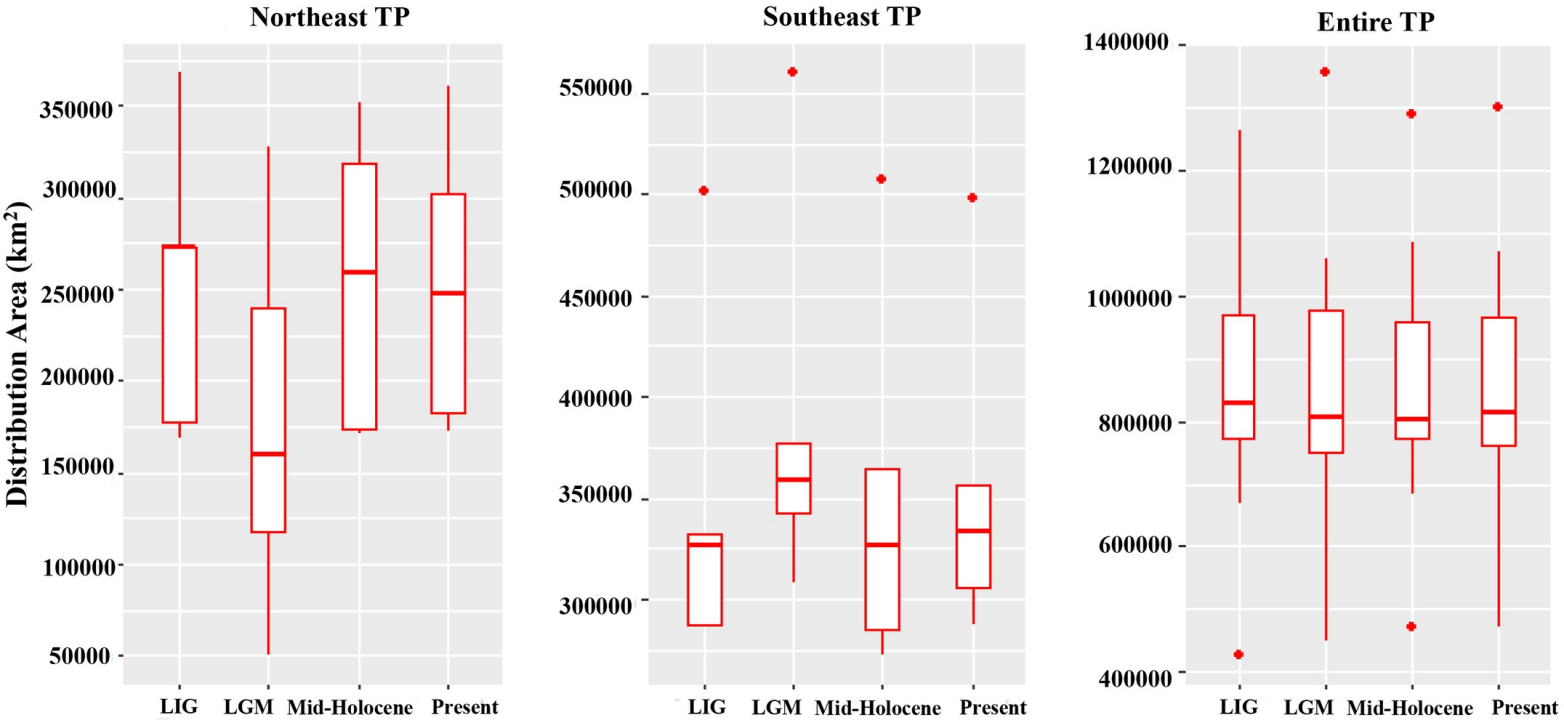
Boxplots of distribution area of plant species in three regions of the TP over four time periods. Three types of range changes were shown: 1) ‘contraction–expansion (northeast)’, 2) ‘expansion–contraction (southeast)’ and 3) ‘stable (entire)’. LIG, the Last Interglacial; LGM, the Last Glacial Maximum.

During the late Quaternary, plant species in three regions of the TP had distinct range dynamics (Fig 2, S2 Fig). For the species in the northeastern part, they mainly presented contraction-expansion scenario before and after the LGM. In contrast, the species in the southeastern TP underwent surprising range expansion during the LGM and then contracted to some extent. For the widespread species across the TP, their distribution exhibited almost no change since the LIG.

**Fig 3:**
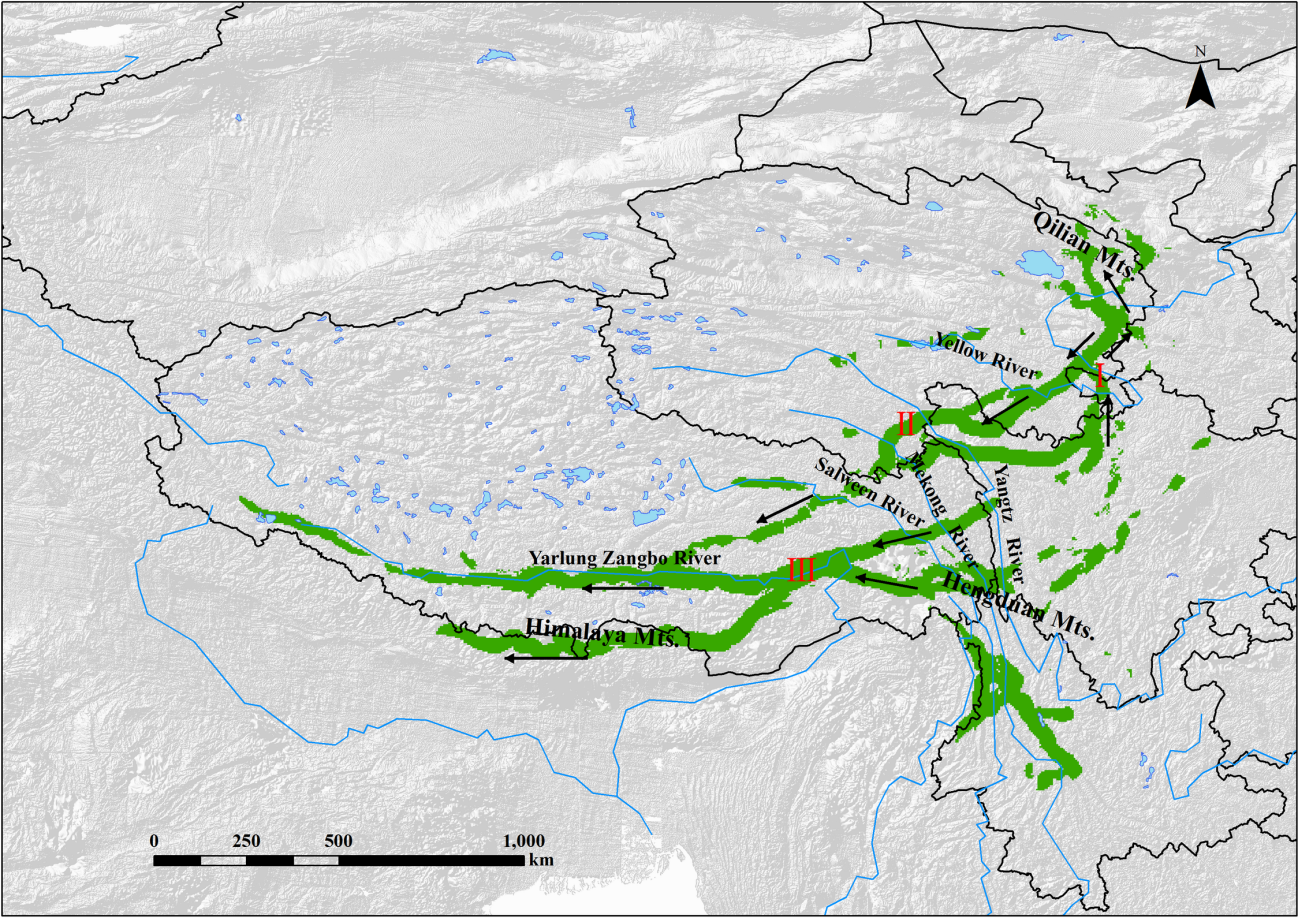
The possible Quaternary dispersal routes of plant species on the Tibetan Plateau. Three pathways were shown: i) the northward route, ii) the southward route, and iii) the westward route. Arrows represent the dispersal direction of species.

Phylogeographical studies have suggested that at least 20 plant species on the TP experienced recolonization during the Quaternary. To date, we have identified the dispersal routes of each plant (S3 Fig) and predicted several major recolonization paths for species on the TP (Fig 3). These dispersal routes are also supported by fossil data and other evidence (summarized in Table 2). We found that at least 3 dispersal corridors existed, and the Hengduan Mountains (HM) seemed to act as the main dispersal source (i.e. glaciation refugia). These paths included: i) the northward path from the northern part of the HM to the Qilian Mountains; ii) the westward path, in which species started in the HM region and recolonized along the Yarlung Zangbo River or Himalaya Mountains; and iii) the southward path, which went from the northeastern part to the southern part throughout the majority of TP’s platform, converged with the Yarlung Zangbo River, and moved further into the vast western region of the TP.

**Table 2:** Fossil and other evidence for supporting the existence of dispersal routes.

Path	Description	Evidence	Source
Westward path (including the Yarlung Zangbo Valley and the Himilaya)	Several oryctocoenoses were found in Namling County and Gyirong Basin;	Fossil	[56, 57]
	The radiation of *Tripterospermum*, *Gentiana* and Adoxaceae from the Hengduan Mountains to the western TP;	Phylogenetic analysis	[34, 58, 59]
	Several species underwent extensive recolonization events from the Hengduan Mountains to Himalaya;	Phylogeographic analysis	[11, 60]
	The great canyon of Yarlung Zangbo River has abundant plants, which is a young floristic region originated from the East Asia flora since the Tertiary;	Floristic analysis	[61]
	The species *Pedicularis longiflora* and *Tsuga dumosa* underwent westward dispersal along this path;	Least-cost path analysis	[16, 62]
	The seed dispersal of *Sophora moorcroftiana* relied on the wind and waterflow in the midstream of Yarlung Zangbo River.	Seed dispersal experiment	[63]
Northward path (including the Hehuang Valley and the Qilian Mountains)	The alternation of grassland and forest in the northeastern TP during the late Quaternary;	Vegetation reconstruction based on pollen	[64, 65]
	The species *Juniperus przewalskii* and *Picea crassifolia* underwent invasion of platform after the last glaciation.	Phylogeographic analysis	[25, 26]
Southward path	The species *Potentilla fruticosa* and *Allium przewalskianum* underwent extensive recolonization events from the northeastern to the southern TP.	Phylogeographic analysis	[22, 23, 34]

## Discussion

### Range dynamics of plants on the TP during the late Quaternary

Within the European and North American continents, contraction-expansion in species distribution was generally predominant [66, 67], owing to the existence of a large and complete ice sheet during the LGM. However, there was no such formation of an ice sheet on the TP [21], and our analysis suggests that the range dynamics of species on the TP were complex during the late Quaternary. It might be argued that these distinct shifts in distribution were associated with the heterogeneous climate and topography across the TP.

In the northeastern TP, the *tabula rasa* scenario seemed to be dominant, which is consistent with previous phylogeographical studies and pollen fossil records [25, 26, 64]. In this region, the climate is colder and drier than that of the southern TP, and the platform is basically flat; only the eastern part has a series of mountain ranges (e.g. the Qilian Mountains and the Minshan Mountains in the northern HM). During the LGM, according to the rare palaeovegetation on the platform and the climatic characteristics [68, 69], we assumed that platform populations of species became extinct and only marginal populations survived within the eastern mountains. Subsequently, when the warmer and wetter Holocene period came, forest and alpine meadow recolonized on the platform [64]. Therefore, the alternation of vegetation on the platform suggests that the LGM might have had greater effects on species distribution in this region.

In the southeastern TP, the species exhibited an expansion-contraction response during the late Quaternary. We assumed that this response was closely related to regional topographic and climatic features. This region is mainly located within the East Himalaya and Hengduan Mountains, which provide ample altitudinal range (e.g. niche) and stable ecological environment [70]. On the other hand, the Indian monsoon produces a warmer and wetter climate compared to other regions of the QTP [31, 71]. The pollen data indicate that the late Quaternary was characterized by a warm temperate climate [72], and phylogeographical studies suggest that this region acted as the major glaciation refugia for many alpine species on the TP [18, 73]. Hence, during the LGM, suitable habitats could have facilitated the persistence of populations in low-elevation areas, and populations on the top of mountains could have moved downward and expanded into other areas because of the abundant niche in this region. In contrast, during the LIG and Holocene period, the warmer and wetter climate would have driven alpine species upward, resulting in their limited distribution in an isolated area.

For the widespread species on the TP, as discussed above, during the late Quaternary, the populations in the northeastern platform may have contracted to refugia, whereas the populations in the southeastern part may have expanded in range. For the populations in the central and southern TP’s platform, a lot of evidence suggests that the LGM had a limited influence on species distribution of the platform [22, 56]. In addition, there might be several microrefugia (e.g. the Three Rivers’ Headstream Regions) within these platform areas [74, 75]. Therefore, as a whole, widely distributed species maintained stable populations throughout the LGM.

### Dispersal routes of plants on the TP

Phylogeographical studies have been able to test which species experienced rapid and extensive range expansion. In this study, using the LCP method, in combination with SDMs and shared haplotypes, we have identified the possible dispersal routes of Tibetan plant species during the Quaternary. Undoubtedly, determining the historical dispersal routes is important to supplement the demographic history of species on the TP.

In this study, we identified three common pathways across the TP. Among the initial locations of these routes, the East Himalaya-Hengduan Mountains seemed to be the dispersal source, which may be associated with this region’s role as the major glaciation refugia [18, 73]. According to historical biogeographic studies, this place was also an important center of origin and radiation of alpine plants, triggering numerous species to migrate into other areas from this region in the Neogene or earlier period [34, 58]. Surprisingly, the migration routes estimated in those studies partially overlapped with the paths during the Quaternary. Thus, it might be argued that species on the TP tend to disperse along several constant routes. We assumed that this could be related to the topographic and monsoon climatic features of the TP. The northward and westward routes identified in this study were basically along the Hehuang Valley and Yarlung Zangbo Valley. Why did species migrate along these valleys? These valleys are not only lower elevation areas (average elevation < 4000 m), but also important vapor channels; in particular, the Yarlung Zangbo Valley is the largest vapor channel of the TP [30]. Hence, with lower topography and strong airflow, these valleys are likely to facilitate the dispersal of seeds and pollen. Additionally, many fossils found in these valleys along with phylogenetic and floristic analysis also support the existence of these corridors (see Table 2). Apart from these two valleys, we predicted that the Yangtze River, Mekong River and Salween River might also be important dispersal pathways. Even not obviously identified in our study, other evidence implied that alpine species would migrate along these valleys during the glacial period [10, 11].

Mountain ridges are another important dispersal corridors, like the Himalaya Mountains in this study, which is a very long-narrow mountainous area. Evidence has shown that the Quaternary glaciations likely had a great impact on plants in this region, resulting in many species becoming extinct [60]. However, because the Himalaya is linked with the Hengduan Mountains, some species could have easily moved to the Himalaya during the postglacial period. In other montane areas around the world, huge mountains (e.g. the Andes) are also regarded as important dispersal corridors [76].

We found that the TP’s platform (average elevation > 4500 m), which has flatter topography, could facilitate long distance dispersal of alpine species, like the southward path in this study. Despite the existence of a series of east-westward mountains across the platform, their relative elevation is actually not so high. Through Barrier analysis, we have also demonstrated that these mountains are not acted as geographical isolations (unpublished data). Thus, several drought-tolerant species (e.g. *Potentilla fruticosa* and *Allium przewalskianum*), originating in the northern TP or adjacent desert (e.g. the Tengger Desert), could migrate into the southern Tibet across the entire TP [22, 23].

## Conclusions

The plant species on the TP exhibited diverse demographic histories during the late Quaternary. There were at least three types of changes in range. Despite the detailed range shifts are distinct across different parts of the TP, they actually had no significant difference, and as a whole, they remained relatively stable status in response to the last glacial, suggesting that LGM had a limited impact on species distribution. In fact, an earlier glaciation (the Naynayxungla glaciation, 0.72–0.50 Ma) might have had a significant influence. After this glaciation, species were continuously dispersed along the low-elevation valleys (e.g. the Yarlung Zangbo Valley and Hehuang Valley), huge mountain ranges (e.g. the Himalaya) or even the flat plateau platform. Under future climate change, the species on the TP could continue to move along these pathways, and thereby these areas should be paid more attention. Besides, the inferences in this study are just based on 20 species, we believe that more diverse range dynamics and dispersal corridors would be found if many species are involved in future studies.

## Supporting information

S1 Table Codes and description for the 19 bioclimatic variables. (DOCX)

S2 Table Number of locality used for modelling the distribution of 20 plant species. (DOCX)

S1 Fig Haplotype patterns of 20 plant species on the Tibetan Plateau. The haplotype compositions of populations for each species were originated form phylogeographical studies (see Table 1). Pie chart corresponds to the sample locations of each species and the diverse colors from green to red are attributed to different chlorotypes or mitotypes.
(RAR)

S2 Fig Range dynamics of 20 plant species over four time periods. LIG, the Last Interglacial; LGM, the Last Glacial Maximum.
(RAR)

S3 Fig Dispersal routes of 20 plant species on the Tibetan Plateau. Warm colors represent higher population connectivity.
(RAR)

## References

[1] Avise JC. Phylogeography: the history and formation of species. Cambridge: Harvard University Press; 2000.

[2] Schönswetter P, Stehlik I, Holderegger R, Tribsch A. Molecular evidence for glacial refugia of mountain plants in the European Alps Mol Ecol. 2005; 14: 3547–3555. doi: 10.1111/j.1365-294X.2005.02683.x 16156822 10.1111/j.1365-294X.2005.02683.x

[3] Hampe A, Arroyo J, Jordano P, Petit RJ. Rangewide phylogeography of a bird-dispersed Eurasian shrub: contrasting Mediterranean and temperate glacial refugia. Mol Ecol. 2003; 12: 3415–3426. 14629356 10.1046/j.1365-294x.2003.02006.x

[4] Hewitt G. The genetic legacy of the Quaternary ice ages. Nature. 2000; 405: 907–913. doi: 10.1038/35016000 10879524 10.1038/35016000

[5] Hewitt G. Genetic consequences of climatic oscillations in the Quaternary. Philos T R Soc B. 2004; 359: 183–195. 10.1098/rstb.2003.1388 PMC1693318 15101575

[6] Tajima F. Statistical method for testing the neutral mutation hypothesis by DNA polymorphism. Genetics. 1989; 123: 585–595. 2513255 10.1093/genetics/123.3.585 PMC1203831

[7] Rogers AR, Harpending H. Population growth makes waves in the distribution of pairwise genetic differences. Mol Biol Evol. 1992; 9: 552–569. 1316531 10.1093/oxfordjournals.molbev.a040727

[8] Fu Y. Statistical tests of neutrality of mutations against population growth, hitchhiking and background selection. Genetics. 1997; 147: 915–925. 9335623 10.1093/genetics/147.2.915 PMC1208208

[9] Phillips SJ, Anderson RP, Schapire RE. Maximum entropy modeling of species geographic distributions. Ecol Model. 2006; 190: 231–259.

[10] Li L, Abbott RJ, Liu B, Sun Y, Li L, Zou J, et al Pliocene intraspecific divergence and Plio-Pleistocene range expansions within *Picea likiangensis* (Lijiang spruce), a dominant forest tree of the Qinghai-Tibet Plateau. Mol Ecol. 2013; 22: 5237–5255. doi: 10.1111/mec.12466 24118118 10.1111/mec.12466

[11] Liu J, Moeller M, Provan J, Gao L, Poudel RC, Li D. Geological and ecological factors drive cryptic speciation of yews in a biodiversity hotspot. New Phytol. 2013; 199: 1093–1108. doi: 10.1111/nph.12336 23718262 10.1111/nph.12336

[12] Wang IJ, Savage WK, Bradley Shaffer H. Landscape genetics and least-cost path analysis reveal unexpected dispersal routes in the California tiger salamander (*Ambystoma californiense*). Mol Ecol. 2009; 18: 1365–1374. doi: 10.1111/j.1365-294X.2009.04122.x 19368644 10.1111/j.1365-294X.2009.04122.x

[13] Li H, Li D, Li T, Qiao Q, Yang J, Zhang H. Application of least-cost path model to identify a giant panda dispersal corridor network after the Wenchuan earthquake-Case study of Wolong Nature Reserve in China. Ecol Model. 2010; 221: 944–952.

[14] Etherington TR, Holland EP. Least-cost path length versus accumulated-cost as connectivity measures. Landscape Ecol. 2013; 28: 1223–1229.

[15] Chan LM, Brown JL, Yoder AD. Integrating statistical genetic and geospatial methods brings new power to phylogeography. Mol Phylogenet Evol. 2011; 59: 523–537. doi: 10.1016/j.ympev.2011.01.020 21352934 10.1016/j.ympev.2011.01.020

[16] Yu H, Zhang Y, Liu L, Qi W, Li S, Hu Z. Combining the least cost path method with population genetic data and species distribution models to identify landscape connectivity during the late Quaternary in Himalayan hemlock. Ecol Evol. 2015; 5: 5781–5791. doi: 10.1002/ece3.1840 26811753 10.1002/ece3.1840 PMC4717335

[17] Qiu Y, Fu C, Comes HP. Plant molecular phylogeography in China and adjacent regions: tracing the genetic imprints of Quaternary climate and environmental change in the world’s most diverse temperate flora. Mol Phylogenet Evol. 2011; 59: 225–244. doi: 10.1016/j.ympev.2011.01.012 21292014 10.1016/j.ympev.2011.01.012

[18] Liu J, Duan Y, Hao G, Ge X, Sun H. Evolutionary history and underlying adaptation of alpine plants on the Qinghai-Tibet Plateau. J Syst Evol. 2014; 52: 241–249.

[19] Wen J, Zhang J, Nie Z, Zhong Y, Sun H. Evolutionary diversifications of plants on the Qinghai-Tibetan Plateau. Front Genet. 2014; 5: 4 doi: 10.3389/fgene.2014.00004 24575120 10.3389/fgene.2014.00004 PMC3921583

[20] Favre A, Päckert M, Pauls SU, Jähnig SC, Uhl D, Michalak I, et al The role of the uplift of the Qinghai-Tibetan Plateau for the evolution of Tibetan biotas. Biol Rev. 2015; 90: 236–253. doi: 10.1111/brv.12107 24784793 10.1111/brv.12107

[21] Li B, Li J. Map of Quaternary glacier on the Qinghai-Xizang (Tibet) Plateau. Beijing: Science Press; 1991.

[22] Sun Y, Ikeda H, Wang Y, Liu J. Phylogeography of *Potentilla fruticosa* (Rosaceae) in the Qinghai-Tibetan Plateau revisited: a reappraisal and new insights. Plant Ecol Divers. 2010; 3: 249–257.

[23] Wu L, Cui X, Milne RI, Sun Y, Liu J. Multiple autopolyploidizations and range expansion of *Allium przewalskianum* Regel. (Alliaceae) in the Qinghai-Tibetan Plateau. Mol Ecol. 2010; 19: 1691–1704. doi: 10.1111/j.1365-294X.2010.04613.x 20345685 10.1111/j.1365-294X.2010.04613.x

[24] Opgenoorth L, Vendramin GG, Mao K, Miehe G, Miehe S, Liepelt S, Liu J, Ziegenhagen B. Tree endurance on the Tibetan Plateau marks the world’s highest known tree line of the Last Glacial Maximum. New Phytol. 2010; 185: 332–342. doi: 10.1111/j.1469-8137.2009.03007.x 19761444 10.1111/j.1469-8137.2009.03007.x

[25] Zhang Q, Chiang T, George M, Liu J, Abbott RJ. Phylogeography of the Qinghai-Tibetan Plateau endemic *Juniperus przewalskii* (Cupressaceae) inferred from chloroplast DNA sequence variation. Mol Ecol. 2005; 14: 3513–3524. doi: 10.1111/j.1365-294X.2005.02677.x 16156819 10.1111/j.1365-294X.2005.02677.x

[26] Meng L, Yang R, Abbott RJ, Miehe G, Hu T, Liu J. Mitochondrial and chloroplast phylogeography of *Picea crassifolia* Kom. (Pinaceae) in the Qinghai-Tibetan Plateau and adjacent highlands. Mol Ecol. 2007; 16: 4128–4137. doi: 10.1111/j.1365-294X.2007.03459.x 17894761 10.1111/j.1365-294X.2007.03459.x

[27] Gao Y, Zhang Y, Gao X, Zhu Z. Pleistocene glaciations, demographic expansion and subsequent isolation promoted morphological heterogeneity: A phylogeographic study of the alpine *Rosa sericea* complex (Rosaceae). Sci Rep-UK. 2015; 5: 11698. 10.1038/srep11698 PMC5155592 26123942

[28] Wan D, Feng J, Jiang D, Mao K, Duan Y, Miehe G, et al The Quaternary evolutionary history, potential distribution dynamics, and conservation implications for a Qinghai-Tibet Plateau endemic herbaceous perennial, *Anisodus tanguticus* (Solanaceae). Ecol Evol. 2016; 7: 1977–1995. 10.1002/ece3.2019 PMC4831433 27099706

[29] Zhang Y, Li B, Zheng D. A discussion on the boundary and area of the Tibetan Plateau in China. Geogr Res. 2002; 21: 1–8.

[30] Lin Z, Wu X. A preliminary analysis about the tracks of moisture transportation on the Qinghai-Xizang Plateau. Geogr Res. 1990; 9: 33–40.

[31] Yao T, Thompson L, Yang W, Yu W, Gao Y, Guo X, et al Different glacier status with atmospheric circulations in Tibetan Plateau and surroundings. Nat Clim Change. 2012; 2: 663–667.

[32] Zhang D, Ye J, Sun H. Quantitative approaches to identify floristic units and centres of species endemism in the Qinghai-Tibetan Plateau, south-western China. J Biogeogr. 2016; 43: 2465–2476.

[33] Wu Z. The flora of Tibet. Beijing: Science Press; 1988.

[34] Matuszak S, Muellner Riehl AN, Sun H, Favre A. Dispersal routes between biodiversity hotspots in Asia: the case of the mountain genus *Tripterospermum* (Gentianinae, Gentianaceae) and its close relatives. J Biogeogr. 2016; 43: 580–590.

[35] Zhang J, Meng S, Rao G. Phylogeography of *Rhodiola kirilowii* (Crassulaceae): A Story of Miocene Divergence and Quaternary Expansion. PLoS ONE. 2014; 9: e112923 doi: 10.1371/journal.pone.0112923 25389750 10.1371/journal.pone.0112923 PMC4229298

[36] Chen S, Wu G, Zhang D, Gao Q, Duan Y, Zhang F, et al Potential refugium on the Qinghai-Tibet Plateau revealed by the chloroplast DNA phylogeography of the alpine species *Metagentiana striata* (Gentianaceae). Bot J Linn Soc. 2008; 157: 125–140.

[37] Zhang X, He X. Phylogeography of *Angelica nitida* (Apiaceae) endemic to the Qinghai-Tibet Plateau based on chloroplast DNA sequences. J Syst Evol. 2013; 51: 564–577.

[38] Zhao C, Ma X, Liang Q, Wang C, He X. Phylogeography of an alpine plant (*Bupleurum smithii*, Apiaceae) endemic to the Qinghai-Tibetan Plateau and adjacent regions inferred from chloroplast DNA sequence variation. J Syst Evol. 2013; 51: 382–395.

[39] Yue L, Chen G, Sun W, Sun H. Phylogeography of *Buddleja crispa* (Buddlejaceae) and its correlation with drainage system evolution in southwestern China. Am J Bot. 2012; 99: 1726–1735. doi: 10.3732/ajb.1100506 23024123 10.3732/ajb.1100506

[40] Zou J, Peng X, Li L, Liu J, Miehe G, Opgenoorth L. Molecular phylogeography and evolutionary history of *Picea likiangensis* in the Qinghai-Tibetan Plateau inferred from mitochondrial and chloroplast DNA sequence variation. J Syst Evol. 2012; 50: 341–350.

[41] Yue L, Sun H. Montane refugia isolation and plateau population expansion: Phylogeography of Sino-Himalayan endemic *Spenceria ramalana* (Rosaceae). J Syst Evol. 2014; 52: 326–340.

[42] Du F, Hou M, Wang W, Mao K, Hampe A. Phylogeography of *Quercus aquifolioides* provides novel insights into the Neogene history of a major global hotspot of plant diversity in south-west China. J Biogeogr. 2017; 44: 294–307.

[43] Duan Y, Gao Q, Zhang F, Li Y, Fu P, Chen S. Phylogeographic analysis of the endemic species *Sibiraea angustata* reveals a marginal refugium in the Qinghai-Tibet Plateau. Nord J Bot. 2011; 29: 615–624.

[44] Chen J, Du Z, Sun S, Gituru RW, Wang Q. Chloroplast DNA phylogeography reveals repeated range expansion in a widespread aquatic herb *Hippuris vulgaris* in the Qinghai-Tibetan Plateau and adjacent areas. PLoS ONE. 2013; 8: e60948 doi: 10.1371/journal.pone.0060948 23565290 10.1371/journal.pone.0060948 PMC3614902

[45] Chen J, Du Z, Yuan Y, Wang Q. Phylogeography of an alpine aquatic herb *Ranunculus bungei* (Ranunculaceae) on the Qinghai-Tibet Plateau. J Syst Evol. 2014; 52: 313–325.

[46] Wang G, He X, Miehe G, Mao K. Phylogeography of the Qinghai-Tibet Plateau endemic alpine herb *Pomatosace filicula* (Primulaceae). J Syst Evol. 2014; 52: 289–302.

[47] Liu Y, Su X, He Y, Han L, Huang Y, Wang Z. Evolutionary history of *Orinus thoroldii* (Poaceae), endemic to the western Qinghai-Tibetan Plateau in China. Biochem Syst Ecol. 2015; 59: 159–167.

[48] Du Z, Wang Q. Allopatric divergence of *Stuckenia filiformis* (Potamogetonaceae) on the Qinghai-Tibet Plateau and its comparative phylogeography with *S*. *pectinata* in China. Sci Rep-UK. 2016; 6: 20883. 10.1038/srep20883 PMC4750007 26864465

[49] Hijmans RJ, Cameron SE, Parra JL, Jones PG, Jarvis A. Very high resolution interpolated climate surfaces for global land areas. Int J Climatol. 2005; 25: 1965–1978.

[50] Brown JL. SDMtoolbox: a python-based GIS toolkit for landscape genetic, biogeographic and species distribution model analyses. Methods Ecol Evol. 2014; 5: 694–700. 10.7717/peerj.4095 PMC5721907 29230356

[51] Veloz SD. Spatially autocorrelated sampling falsely inflates measures of accuracy for presence-only niche models. J Biogeogr. 2009; 36: 2290–2299.

[52] Hijmans RJ. Cross-validation of species distribution models: removing spatial sorting bias and calibration with a null model. Ecology. 2012; 93: 679–688. 22624221 10.1890/11-0826.1

[53] Boria RA, Olson LE, Goodman SM, Anderson RP. Spatial filtering to reduce sampling bias can improve the performance of ecological niche models. Ecol Model. 2014; 275: 73–77.

[54] R Core Team. R: a language and environment for statistical computing. Vienna: R Foundation for Statistical Computing, 2016; https://www.R-project.org/.

[55] Vignieri SN. Streams over mountains: influence of riparian connectivity on gene flow in the Pacific jumping mouse (*Zapus trinotatus*). Mol Ecol. 2005; 14:1925–1937. doi: 10.1111/j.1365-294X.2005.02568.x 15910316 10.1111/j.1365-294X.2005.02568.x

[56] Shi Y, Li J, Li B. Uplift and environmental changes of Qinghai-Xizang (Tibetan) in the Late Cenozoic. Guangzhou: Guangdong Science and Technology Press; 1998.

[57] Song Z, Liu G. Early Tertiary palynofloral and its significance of palaeogeography from northern and eastern Xizang. Beijing: Science Press; 1982.

[58] Ho T, Liu S. A worldwide monograph of *Gentiana*. Beijing: Science Press; 2001.

[59] Liang H, Wu Z. On the Taxonomic system, phylogeny and distribution in Adoxaceae. Acta Bot Yunn. 1995; 17: 380–390.

[60] Cun Y, Wang X. Plant recolonization in the Himalaya from the southeastern Qinghai-Tibetan Plateau: Geographical isolation contributed to high population differentiation. Mol Phylogenet Evol. 2010; 56: 972–982. doi: 10.1016/j.ympev.2010.05.007 20472080 10.1016/j.ympev.2010.05.007

[61] Sun H, Zhou Z. The characters and origin of the flora from the big bend gorge of Yalutsangpu (Brahmabutra) River, eastern Himalayas. Acta Bot Yunn. 1996; 18: 185–204.

[62] Yu H, Zhang Y, Li S, Qi W. Predicting the dispersal routes of alpine plant *Pedicularis longiflora* (Orobanchaceae) based on GIS and species distribution models. Chin J Appl Ecol. 2014; 25: 1669–1673. 25223022

[63] Liu Z, Zhao W, Li Z. Characteristics of the seed bank of *Sophora moorcroftiana* population in the middle reach of Yarlung Zangbo River, Tibet. Acta Ecol Sin. 2001; 22: 715–722.

[64] Tang L, Shen C, Kong Z, Wang F, Liu K. Pollen evidence of climate during the Last Glacial Maximum in eastern Tibetan plateau. J Glaciol Geocryol. 1998; 20: 133–140.

[65] Wan H, Tang L, Zhang H, Li C, Pang Y. Pollen record reflects climate changes in eastern Qaidam Basin during 36–18 ka B.P. Quat Sci. 2008; 28: 112–121.

[66] Taberlet P, Fumagalli L, Wust Saucy AG, Cosson JF. Comparative phylogeography and postglacial colonization routes in Europe. Mol Ecol. 1998; 7: 453–464. 9628000 10.1046/j.1365-294x.1998.00289.x

[67] Soltis DE, Morris AB, McLachlan JS, Manos PS, Soltis PS. Comparative phylogeography of unglaciated eastern North America. Mol Ecol. 2006; 15: 4261–4293. doi: 10.1111/j.1365-294X.2006.03061.x 17107465 10.1111/j.1365-294X.2006.03061.x

[68] Tang L, Shen C. Late Cenozoic vegetational history and climatic characteristics of Qinghai-Xizang Plateau. Acta Micropalaeontol Sin. 1995; 13: 321–338.

[69] Yu G, Chen X, Ni J, Cheddadi R, Guiot J, Han H, et al Palaeovegetation of China: a pollen data-based synthesis for the mid-Holocene and last glacial maximum. J Biogeogr. 2000; 27: 635–664.

[70] Yan Y, Yang X, Tang Z. Patterns of species diversity and phylogenetic structure of vascular plants on the Qinghai-Tibetan Plateau. Ecol Evol. 2013; 3: 4584–4595. doi: 10.1002/ece3.847 24340197 10.1002/ece3.847 PMC3856756

[71] An Z, Kutzbach JE, Prell WL, Porter SC. Evolution of Asian monsoons and phased uplift of the Himalaya-Tibetan plateau since Late Miocene times. Nature. 2001; 411: 62–66. doi: 10.1038/35075035 11333976 10.1038/35075035

[72] Harrison SP, Yu G, Takahara H, Prentice IC. Palaeovegetation (Communications arising): diversity of temperate plants in east Asia. Nature. 2001; 413: 129–130. doi: 10.1038/35093166 11557970 10.1038/35093166

[73] Yu H, Zhang Y. Advances in phylogeography of alpine plants in the Tibetan Plateau and adjacent regions. Acta Bot Boreali-Occident Sin. 2013; 33: 1268–1278.

[74] Gao Q, Zhang D, Duan Y, Zhang F, Li Y, Fu P, et al Intraspecific divergences of *Rhodiola alsia* (Crassulaceae) based on plastid DNA and internal transcribed spacer fragments. Bot J Linn Soc. 2012; 168: 204–215.

[75] Zhang F, Gao Q, Zhang D, Duan Y, Li Y, Fu P, et al Phylogeography of *Spiraea alpina* (Rosaceae) in the Qinghai-Tibetan Plateau inferred from chloroplast DNA sequence variations. J Syst Evol. 2012; 50: 276–283.

[76] Luebert F, Weigend M. Phylogenetic insights into Andean plant diversification. Front Ecol Evol. 2014; 2: 27.

