# Supplementary material for: Diverse range dynamics and dispersal routes of plants on the Tibetan Plateau during the late Quaternary
Source: PLoS One. 2017 May 5;12(5):e0177101. doi: 10.1371/journal.pone.0177101 (PMC5419580; doi:10.1371/journal.pone.0177101)
Supplement: S1 Table — (DOCX) [file pone.0177101.s001.docx]

**S1 Table. Codes and description for the 19 bioclimatic variables.**

| Code | Description | Code | Description |
| --- | --- | --- | --- |
| Bio1 | Mean annual temperature | Bio11 | Mean temperature of coldest quarter |
| Bio2 | Mean diurnal range (Mean of monthly (max temp - min temp)) | Bio12 | Annual precipitation |
| Bio3 | Isothermality (Bio2/Bio7) (*100) | Bio13 | Precipitation of wettest month |
| Bio4 | Temperature seasonality (standard deviation *100) | Bio14 | Precipitation of driest month |
| Bio5 | Max temperature of warmest month | Bio15 | Precipitation seasonality (Coefficient of Variation) |
| Bio6 | Min temperature of coldest month | Bio16 | Precipitation of wettest quarter |
| Bio7 | Temperature annual range (Bio5-Bio6) | Bio17 | Precipitation of driest quarter |
| Bio8 | Mean temperature of wettest quarter | Bio18 | Precipitation of warmest quarter |
| Bio9 | Mean temperature of driest quarter | Bio19 | Precipitation of coldest quarter |
| Bio10 | Mean temperature of warmest quarter |  |  |
