# Supplementary material for: Diverse range dynamics and dispersal routes of plants on the Tibetan Plateau during the late Quaternary
Source: PLoS One. 2017 May 5;12(5):e0177101. doi: 10.1371/journal.pone.0177101 (PMC5419580; doi:10.1371/journal.pone.0177101)
Supplement: S2 Table — (DOCX) [file pone.0177101.s002.docx]

**S2 Table. Number of locality used for modelling the distribution of 20 plant species.**

| Species | No. | Species | No. |
| --- | --- | --- | --- |
| *Juniperus przewalskii* | 24 | *Potentilla fruticosa* | 62 |
| *Picea crassifolia* | 35 | *Allium przewalskianum* | 55 |
| *Metaentiana striata* | 25 | *Sibiraea angustata* | 59 |
| *Angelica nitida* | 23 | *Hippuris vulgaris* | 54 |
| *Bupleurum smithii* | 40 | *Ranunculus bungei* | 50 |
| *Buddleja crispa* | 29 | *Pomatosace filicula* | 31 |
| *Picea likiangensis* | 46 | *Orinus thoroldii* | 30 |
| *Taxus wallichiana* | 49 | *Rosa sericea* | 65 |
| *Spenceria ramalana* | 31 | *Stuckenia filiformis* | 57 |
| *Quercus aquifolioides* | 63 | *Anisodus tanguticus* | 36 |
